# Population mixing and childhood leukaemia: Fallon and other US clusters

**DOI:** 10.1038/sj.bjc.6601982

**Published:** 2004-06-29

**Authors:** L Kinlen, R Doll

**Affiliations:** 1 Cancer Epidemiology Unit, Oxford University, Gibson Building, Radcliffe Infirmary, Oxford OX2 6HE, UK; 2 Clinical Trials Service Unit, Oxford University, Harkness Building, Radcliffe Infirmary, Oxford OX2 6HE, UK

Cancer clusters – distinctive geographical groupings of cases – have always attracted attention and none more so than those involving childhood leukaemia. The public, however, has often found the approach of epidemiologists to these clusters disappointing. For the frequent uncertainty about the genuineness of a cluster in reflecting a raised underlying risk, and its treatment only as generating a hypothesis, with consequently greater interest in possible future cases than in the (seemingly definite) recent past, can all appear unhelpful. The fact remains that the choice of boundaries in space and time greatly influences the magnitude of observed excesses; and tests of significance are strictly inappropriate without a prior hypothesis, since a chance would inevitably from time to time generate extreme fluctuations in disease occurrence, which, in the absence of further information, cannot be distinguished from a genuinely raised risk. The population mixing hypothesis of childhood leukaemia (Kinlen, 1988) moved attention away from aggregations attracting notice and on to a possible cause; if correct, it should reveal significant excesses even when these were previously unsuspected.

If the long-standing suspicion of an infective origin in childhood leukaemia is correct, it must (like all known infection-linked cancers) belong to that large group of illnesses that are rare responses to the relevant infection, otherwise marked space–time clustering would be more evident. This underlying infection, therefore, would, like polio virus infection, be mainly immunizing and subclinical. The population mixing hypothesis originated in the raised levels of childhood leukaemia near the nuclear sites at Sellafield in north-west England and Dounreay in northern Scotland, which could not be explained in terms of radiation exposure; for these remote and isolated rural areas had experienced highly unusual population movements (Kinlen, 1988, 2000). From the well-established premise that epidemics depend upon the presence of sufficient numbers of susceptible individuals and that these are more prevalent in rural areas because of the reduced opportunities for contacts with a wider infective pool, it was argued that a localised epidemic of an underlying infection would be promoted by large-scale rural–urban population mixing (i.e. by the increased level of contacts between susceptible and infected individuals) (Kinlen, 1988). High doses of an infective agent are more likely to be received in an epidemic than in sporadic infection and, by analogy to leukaemia in cats, these may produce a heightened risk of leukaemia.

From this idea began a series of studies that eventually covered all known examples of extreme rural–urban mixing in Britain in the past 60 years, each of which revealed a significant temporary excess (Kinlen, 1995). They included rural new towns (Kinlen *et al*, 1990), wartime evacuation of children to rural areas (Kinlen and John, 1994), rural inflows of servicemen in the early days of national military service (Kinlen and Hudson, 1991), areas near large rural (non-nuclear) construction sites (Kinlen *et al*, 1995), rural Scottish communities where a large proportion of men worked away from home in the North Sea oil industry (Kinlen *et al*, 1993) and wartime Orkney and Shetland where large numbers of servicemen were stationed (Kinlen and Balkwill, 2001). In the last four studies, the incomers were all adults, indicating that the infection is not confined to children. Studies of unusual patterns of contact outside Britain have confirmed their importance in relation to childhood leukaemia (Kinlen and Petridou, 1995; Petridou *et al*, 1996; Alexander *et al*, 1997, 1999; Koushik *et al*, 2001; Boutou *et al*, 2002).

In the first US study of this subject, leukaemia incidence in SEER registry data was recently examined in more rural counties that had experienced the largest population influxes (Wartenberg *et al*, 2004). The use of whole counties as the basic geographic unit of study would have reduced the likelihood of finding positive evidence: in Britain, except for the extensive postwar construction of hydroelectric schemes in the Scottish Highlands, these excesses are not apparent at the county level, but only in the (smaller) local authority areas or parishes most exposed to the mixing. Despite this potential obstacle, however, this US study found some evidence of population mixing effects on childhood leukaemia.

The association with population mixing of a pronounced cluster of childhood leukemia when noticed outside a formal study, when each is extreme in degree, must also be relevant here. An example is the most well-known (until recently) US cluster, in Niles, Illinois, involving eight cases in 1957–1960 centred on a crowded parish school, occurred when the town received a massive influx of new residents, although its possible significance was not recognised at the time; in the decade 1950–1960, its population increased by 5.6-fold from 3587 to 20 393 with much of the influx in 1955–1960 into the relevant parish (Heath and Hasterlick, 1963). Niles is suburban rather than rural, but the variety of origins of incoming residents may have made for differences in proportions of susceptibles within subgroups (as may have occurred in English towns with marked increases in commuting levels, for these also experienced excesses (Kinlen *et al*, 1991)). The findings of their detailed study of the Niles cases (Heath and Hasterlick, 1963) led the Centre for Disease Control to create a special leukaemia section to investigate clusters, but overall with disappointing results.

However, the magnitude of the excess in Niles is dwarfed by that in rural (largely desert) Churchill County, Nevada where 10 cases of childhood leukaemia were diagnosed in only 2 years (eight in 2000, two in 2001) compared to less than one expected; these cases were mainly in the small town of Fallon (population 7536 in 2000) and they have aroused widespread concern and interest. Indeed, no more striking childhood leukaemia cluster in the world has been traced (Alexander, 1993) and its extreme nature even at the county level (*P*=4.3 × 10^−9^) has recently been demonstrated (Steinmaus *et al*, 2004).

The efforts of the Nevada State Health Department to address earlier recommendations have recently been reviewed in a long-awaited final report by a specialist panel (Expert Panel on Childhood Leukemia in Churchill County, Nevada, 2004; Sinks and Smith, 2004). No evidence was found to suggest that the cluster was due to any environmental contaminant, including arsenic levels in water and jet fuel emissions from planes of the nearby naval air station.

The only putative cause which the panel could not exclude was the effect of the recent large increase in the numbers of military personnel temporarily assigned to the Fallon Naval Air Station for training, reaching the extraordinary level of 55 000 in 2000 from 20 000 per year in the early 1990s (U.S. Navy, 2002; GlobalSecurity.org, 2003). However, the panel could not decide whether the temporariness of the trainees' residence made them relevant to this hypothesis. This is surprising since infective transmissions, or even epidemics, are not usually regarded as requiring more than a short period of exposure. In fact, except for rural new towns, much of the support for the hypothesis has come from situations, like that near this naval air base, with no major increase in the permanent population, but which involved large temporary influxes and much ‘changing of places’. Thus, excesses of childhood leukaemia were found near rural military camps when the numbers of national servicemen increased in the early 1950s (Kinlen and Hudson, 1991). Such excesses also occurred near large rural construction projects in Britain (Kinlen *et al*, 1993, 1995) where the large-scale coming and going of men carrying out different jobs is typical. Thus, the building of the THORP plant at Sellafield in the years 1984–1993 involved a workforce of 50 000, although the maximum working at any one time was 7800.

Further, the expert panel assumed that the relevant infection would have been introduced into the community when trainees were for the first time stationed in the area, producing an epidemic occurring among local children in that earlier period. This is too simple a model of the probable history of the infection. The dynamics of epidemics are complex with much evidence of threshold effects and they do not usually begin with the first opportunity for transmission of an infection, but only when a complex set of circumstances has occurred (Topley 1942; Anderson and May, 1991). Previous excesses of childhood leukaemia associated with population mixing have not begun with first exposure to incomers but only when their numbers reached fairly high levels. Thus, the excess linked to the construction of the Sullom Voe oil terminal in Shetland began not in the mid-1970s when its construction was started but in 1979 when the work force and its turnover on the site reached unusually high levels (Kinlen *et al*, 1993). It would therefore not be predicted, nor is it found, that excesses would occur near military bases in general (Kinlen and Hudson, 1991; Steinmaus *et al*, 2004), but only near that minority with marked increases of personnel.

At Fallon, any epidemic would initially be among the trainees coming from various parts of the US, whose previous experience of infective agents can hardly have been uniform. An epidemic would have been promoted among these personnel as numbers, population density and contacts (both direct and indirect) increased on the base in the late 1990s. The variety of origins of servicemen and their relatively crowded conditions combine to promote infective transmission and on occasions epidemics, and it is hardly surprising that military camps and bases both in the US and other countries have figured prominently in the history of infectious disease epidemiology since at least the First World War (Love and Davenport, 1919). The spread of an epidemic of the underlying infection to local residents would then occur secondarily with consequent increases of its complication, childhood leukaemia, as also noted for paralytic poliomyelitis near military camps when personnel numbers greatly increased (Kinlen and Hudson, 1991).

There is no shortage of routes (including schools and civilian workers at the bases) for epidemics to affect more permanent residents of local areas. Fallon residents may reasonably be assumed to include a relatively high proportion of ‘susceptibles’: for its desert location would tend to prevent or delay some of its residents from acquiring the same experience of infective agents as some trainees, drawn as they were from all parts of the country. Fallon's earlier indirect exposure to lower numbers of trainees on the air base, which the panel stressed, would not prevent an epidemic in the late 1990s when the annual trainee numbers became massive, if some ‘contact threshold level’ was exceeded. It is also notable that some children with leukaemia were not even present in that earlier period and for them, exposure to (the exceptional levels of) trainees in the late 1990s could only have been recent (no less than four of them had been resident for less than 3 years before diagnosis; no details were provided for the other six). The occurrence of the last case in December 2001 would be in keeping with a decline in the numbers of susceptible residents to below some threshold level for the relevant underlying infection.

The indirect exposure of Fallon in only a few years to around 100 000 people from outside the area represents a more extreme example of rural–urban population mixing than any of those traced and studied in Britain. That the world's most sharply defined cluster of childhood leukaemia should occur in association with the most extreme example of rural–urban population mixing so far recorded could not be more arresting. Given the support that has accumulated from earlier tests of the hypothesis, strong reasons would be required for rejecting a link.

Among its recommendations, the panel urged that new hypotheses about the causation of childhood leukaemia be proposed and that opportunities then be found for testing them. However, this already implies a turning away from the Fallon cluster and from population mixing, which they had noted as being of possible relevance. It would be unfortunate if interest was abandoned in population mixing in relation to this cluster before its relevance had been thoroughly considered. Indeed, details were not tabulated in the report for all the cases on such elementary aspects as age at diagnosis (and calender year), length of residence in the area before diagnosis and parental occupation. It may be hoped that further work will include a detailed search for the underlying agent in the blood samples that have been collected and stored from the affected young people.
